# rbpCNN: a biophysics-informed deep learning model for predicting piRNA and mRNA interactions

**DOI:** 10.1038/s41598-026-48797-5

**Published:** 2026-04-13

**Authors:** Ahmet Gürhanlı, Sajjad Nematzadeh, Taner Çevik, Amir Seyyedabbasi, Fatih Şahin, Jawad Rasheed

**Affiliations:** 1 Department of Computer Engineering, Faculty of Engineering and Natural Sciences, Istanbul Topkapi University, Istanbul, Turkey; 2 Department of Software Engineering, Faculty of Engineering and Natural Sciences, Istanbul Topkapi University, Istanbul, Turkey; 3 Department of Computer Engineering, Faculty of Engineering and Natural Sciences, Istanbul Rumeli University, Istanbul, Turkey; 4 Computer Engineering Department, Istinye University, Istanbul, Turkey; 5 Department of Computer Engineering, Istanbul Sabahattin Zaim University, Istanbul, 34303 Turkey; 6 Department of Software Engineering, Istanbul Nisantasi University, Istanbul, 34398 Turkey; 7 Research Institute, Istanbul Medipol University, Istanbul, 34810 Turkey; 8 Applied Science Research Center, Applied Science Private University, Amman, Jordan

**Keywords:** RNA interaction prediction, Deep learning, Convolutional neural networks, Computational biology and bioinformatics, Mathematics and computing

## Abstract

Predicting the interactions between piRNA sequences and mRNA sequences is central to understanding post-transcriptional regulation in the germline and to the design of perturbations that modulate PIWI (P-element-induced wimpy testis) guided silencing. Any improvement in the accuracy of predictions about RNA sequence interactions is vital to the enhancements that can be made in many critical medical fields. This paper proposes rbpCNN, a lightweight convolutional neural network (CNN) that augments nucleotide-pair encoding with biophysically motivated interaction channels prior to learning. By adding one compatibility channel, two helix-run channels, one positional channel, and one structural channel, we aimed to support the predictions of the CNN layer and improve prediction accuracy. The resulting network is lightweight and achieves strong performance relative to existing solutions on this benchmark. Experimental results showed that rbpCNN achieves an AUC (area under the receiver operating characteristic curve) of 96.55% and an accuracy of 90.66% in fivefold validation, and an AUC of 94.19% and an accuracy of 86.74% on a separate, fully independent external dataset, with performance competitive with and, in several metrics, exceeding previously reported results on the same benchmark.

## Introduction

PIWI-interacting RNAs (piRNAs) direct PIWI proteins to suppress transposons and control transcripts in the germline and some somatic cells^1^. Unlike miRNAs, piRNAs vary more in length, originate from different biogenesis pathways (primary/phased and ping‑pong), and can function through endonucleolytic “slicing” and effects on chromatin^2^. These distinctions make computational prediction more complex, as rules for complementarity and mismatch tolerance depend on the PIWI protein, the species, and additional factors^3^. Recent reviews provide comprehensive overviews of this pathway and its mechanistic diversity^4^.

In C. elegans, PRG‑1 CLASH and subsequent studies have shown that piRNA binding is more common in coding regions and follows distinct complementarity patterns that differ from traditional miRNA seed rules^5^. Wu et al. performed a transcriptome-wide analysis of piRNA binding sites in C. elegans and showed that piRNA targeting is broadly distributed, with notable enrichment in coding regions, highlighting the importance of positional context in piRNA–mRNA interaction modeling^6^. Recent research also refines our understanding of the mechanism in mammals: the cofactor GTSF1 significantly boosts PIWI slicing activity and relaxes the strictness of complementarity requirements^7^. This helps explain how weak guide–target pairings can still result in effective cleavage^8^. Overall, these findings indicate that accurate prediction models may benefit from incorporating sequence details^9^, positional factors, and ‘biophysics-aware’ features rather than relying solely on seed matching. Izumi et al.^10^ showed that the two silkworm Gtsf paralogs, Gtsf1 and Gtsf1L, selectively activate their partner PIWI proteins and thereby promote piRNA-guided target cleavage and piRNA amplification.

Lu et al. generated an in vivo RNA duplex map and showed that transcriptome-wide RNA–RNA interactions can reveal higher-order RNA structural organization in living cells^11^. Broader RNA duplex-mapping methods, including transcriptome-wide RNA–RNA interaction mapping^12^ and hiCLIP-based approaches^13^, have further expanded our understanding of RNA base-pairing and higher-order RNA structures. Additionally, RIL‑seq has been used to detect sRNA–mRNA networks in bacteria^14^. Collectively, these techniques show that features like non-seed pairing, G:U wobble pairs, bulges, and long‑range interactions are common, highlighting their importance in modeling RNA interactions.

HITS-CLIP and PAR-CLIP initially facilitated the creation of transcriptome-wide maps of AGO and RBP binding sites; eCLIP^15^ further standardized and expanded this framework to encompass hundreds of RBPs (ENCODE), thereby providing robust positive and negative examples for analytical learning^16^. For purposes of supervision and benchmarking within the small-RNA domain, community resources such as miRTar-Base^17^ and ENCORI/starBase^18^ compile experimentally validated interactions alongside sites supported by CLIP evidence.

Before deep learning, target prediction depended on combinations of complementarity scoring, thermodynamics, and accessibility^19^. Krüger and Rehmsmeier^20^ presented RNAhybrid as a fast and flexible microRNA target prediction tool that identifies energetically favorable small RNA–mRNA duplexes and supports seed-region constraints for more adaptable target-site analysis. General RNA–RNA tools^21^ expanded this with explicit opening energies (RNAup), fast heuristics (RNAplex), seed‑and‑extend with accessibility (IntaRNA), suffix‑array acceleration (RIsearch2), and accurate folding thermodynamics^22^ (ViennaRNA 2.0)^23^. Conceptual reviews outlined non‑seed contributions that inspired more advanced features^24^.

End-to-end neural models started with deepTarget^25^, miRAW^26^, and DeepMirTar^27^, then evolved into hybrid CNN/RNN or transformer versions like miRBind^28^, miTAR^29^, TEC-miTarget^30^ and DMISO^31^. These models usually learn interaction patterns from miRNA–mRNA chimeras or reporter assays, often combining sequence and structure information. However, most of these focus on miRNAs (AGO) rather than PIWI/piRNAs, and therefore they capture different rules.

Yang et al.^32^ proposed a deep learning framework that combines convolutional feature extraction with multi-head attention to predict piRNA–mRNA interactions by capturing sequence motifs and implicit binding relationships. A recent study, rbpTransformer, used the same data sets and employed a transformer-based deep learning model for predicting piRNA and mRNA interactions and evaluated various design alternatives to improve accuracy and AUC results^33^.

When it comes to piRNAs, the available community resources have significantly advanced. For example, piRBase^34^ (versions 2 and 3) offers curated catalogs and annotations for multiple species, making it easier to access comprehensive data^35^. piRNAclusterDB^36^ provides a summary of cluster locations across more than 50 species, giving researchers a valuable overview. Additionally, pirScan^37^ applies C. elegans-specific rules to identify target sites and suggest silent mutations that can help prevent germline silencing. Recent analyses covering whole transcriptomes highlight a strong preference for binding in the CDS regions in vivo, drawing attention to the differences between predictions based solely on sequence and actual experimental findings.

In recent years, several advanced computational frameworks have been introduced to address disease-associated prediction problems across diverse bioinformatics settings, reflecting the increasing adoption of deep and hybrid learning paradigms. scATD^38^ introduces an adaptive transfer-learning and knowledge-distillation framework that leverages large language models to enable high-throughput, patient-agnostic drug sensitivity prediction from single-cell RNA-sequencing data, achieving improved accuracy, generalization, efficiency, and interpretability without requiring dataset-specific model fine-tuning. scTSNN^39^ proposes a tensor-based shared nearest neighbor anchor clustering framework that leverages local–global topological structure learning and density-based anchor detection to achieve accurate, robust, and fully unsupervised cell-type identification and structure discovery in single-cell RNA-sequencing data. circRDRP^40^ introduces a disease-aware hybrid graph neural network framework that integrates circRNA, drug, and disease information to improve the prediction of circRNA-mediated drug resistance, demonstrating enhanced accuracy and robustness for precision oncology applications. DSRNAFold^41^ presents a phased learning framework that integrates RNA sequence and structural context with pairing constraints to improve the robustness and accuracy of RNA secondary structure prediction, particularly for modeling long-range interactions and pseudoknots. Collectively, these studies underscore the effectiveness of incorporating domain-specific priors and advanced learning architectures for disease-associated biological prediction, while addressing problem settings distinct from piRNA–mRNA interaction modeling.

Recent RNA-focused learning methods have increasingly incorporated structured biological context beyond plain sequence encoding. For example, Wang et al. proposed a noise-consistent hypergraph autoencoder for cancer ceRNA association prediction, emphasizing robust structure-aware representation learning in regulatory networks^42^. Wang et al. also introduced a dynamic multi-scale hypergraph learning framework^43^ and, more recently, the HpMiX framework^44^, both of which further illustrate the value of multiscale and topology-informed modeling in RNA-related prediction tasks. Although these methods address ceRNA/disease association rather than direct piRNA–mRNA interaction prediction, they reflect a broader trend toward biologically structured representation learning. In contrast, rbpCNN focuses on local interaction modeling through an explicit multi-channel piRNA–mRNA tensor enriched with pairing- and structure-informed priors.

In the suggested rbpCNN model, each piRNA-mRNA pairing is initially transformed into a multi-channel interaction tensor that fuses traditional nucleotide-pair identity channels with other biophysically relevant channels that account for compatibility, helix continuity, positional preference, and structural availability. This tensor is then processed by a streamlined CNN backbone to generate a binding probability, and Grad-CAM is employed to highlight important areas on the interaction grid that influence the prediction.

In contrast to miRNA-focused CNNs, rbpCNN specifically incorporates Watson–Crick and G:U pairing, includes run-length indicators for contiguous helix formation, integrates positional biases identified in CLASH studies, and reduces the weight of nucleotides estimated to be structurally unavailable.

The main contributions of this work are summarized as follows:

- We propose a biophysics-informed multi-channel interaction tensor that encodes base-pairing identity, compatibility priors, helix-run signals, positional information, and structural accessibility for piRNA–mRNA interaction modeling.
- We develop a lightweight CNN architecture that operates directly on this tensor, enabling efficient learning of local interaction patterns.
- We provide a unified evaluation framework comparing rbpCNN with representative methods under consistent training and testing conditions.
- We perform interpretability analysis using Grad-CAM, highlighting biologically meaningful interaction regions.
- We demonstrate that integrating structured biophysical priors with deep learning improves predictive performance while maintaining model simplicity.

The novelty of rbpCNN lies not in any single biophysical heuristic alone, but in the structured integration of multiple priors into a unified interaction tensor and its direct use in a lightweight CNN framework. As depicted in Table 1, rbpTransformer^33^ improves over Yang et al.^32^ by explicitly modeling inter-sequence relationships through cross-attention, whereas Yang et al. relies on attention over independently processed sequence features. In contrast, rbpCNN further advances this direction by encoding interactions directly in the input space via a structured multi-channel interaction tensor.

**Table 1:** Method comparison.

Method	Input representation	Biophysical priors	Model architecture	Interaction modeling	Interpretability	Independent test
Yang et al. (2021)^32^	Separate one-hot encoded sequences	None	CNN + SE + multi-head attention	Implicit via attention	Attention maps	Yes
rbpTransformer^33^	k-mer based sequence encoding (integer + positional encoding)	None (implicit only)	Transformer (self-attention + cross-attention, multi-core)	Explicit cross-attention between sequences	Attention weights / maps	No (cross validation)
Classical tools (RNAhybrid, pirScan)	Sequence alignment / thermodynamic rules	Strong (explicit)	Analytical / scoring	Explicit pairing rules	Limited	Yes
rbpCNN (this work)	Multi-channel interaction tensor (pairwise representation)	Explicit structured priors (21 channels)	Lightweight CNN	Explicit pairwise interaction modeling (input-level)	Grad-CAM on interaction maps	Yes

Unlike Yang et al. and rbpTransformer, which learn interaction patterns through attention mechanisms, rbpCNN explicitly encodes these interactions at the input level, enabling the direct learning of biologically meaningful pairing patterns with a simpler architecture.

## Materials and methods

A biophysics-informed interaction tensor is constructed by augmenting the 16 base-pair channels with five biologically motivated channels (compatibility, helix contiguity in two directions, relative position, and structural availability). These properties are coded into 5 extra channels and concatenated with the original 16 channels that encode the intersection type, which can be one of the possible 16 pairs, which are AA, AC, …, TT.

We will first explain dataset acquisition, then present the data flow of the AI model, and briefly discuss the role of each deep learning component. Then, the tensor resulting from the concatenation of the channels driven by input sequences will be discussed. In a section, the mathematical model of the solution will be presented. Finally, we will focus on the core algorithms that are used in the AI model.

### Dataset acquisition

In this study, we used two datasets, WT CLASH and CSR-1CLASH, which are derived from wild-type PRG-1 CLASH experiments in C. elegans by Shen et al.^45^. These datasets were used by Yang et al.^32^ to develop a deep learning solution and are available online for further research.

#### WT CLASH dataset

Following Yang et al.^32^, the primary training and validation data were derived from wild-type C. elegans CLASH experiments^45^ focusing on the Argonaute protein PRG-1. Chimeric reads were first separated into piRNA and mRNA segments. The piRNA segment was required to perfectly match a known piRNA annotated in WormBase (WS275). The remaining mRNA segment was then mapped to the C. elegans transcriptome, and the mapped region was extended by ± 15 nucleotides to account for possible RNase trimming during the CLASH protocol.

To construct a high-confidence positive set, Yang et al.^32^ applied several stringent filters:

(i) at least seven supporting chimera reads,
(ii) mRNA segment length of at least 14 nucleotides,
(iii) no mismatches in the alignment,
(iv) removal of non-uniquely mapped sites across isoforms, and
(v) negative piRNA–target binding energy as estimated by RNAup.

After these filtering steps, the final positive set contained 60,438 high-confidence piRNA–mRNA interactions, involving 9,397 distinct mRNA targets and 7,126 piRNAs.

To generate the corresponding negative set, a loose binding set (LBS) was first constructed using more relaxed criteria (read count > 0, segment length ≥ 7 nucleotides, and up to one mismatch). For each piRNA, candidate negatives were then sampled from mRNAs not included in the LBS. Candidate pairs overlapping with the positive set were removed, and only pairs with predicted binding energy below zero were retained. This procedure produced 60,438 negative piRNA–mRNA pairs.

Importantly, these negatives were not intended to represent arbitrary random sequence pairs, but rather plausible non-interacting candidates drawn from the same overall experimental context. This design makes the prediction task more challenging and biologically relevant by reducing the likelihood of trivially separable negatives. At the same time, we acknowledge that a negative construction strategy based on outside-LBS selection and energy-based filtering may still introduce some degree of distributional bias. For this reason, we explicitly report descriptive comparisons between the positive and negative sets in terms of sequence length, GC content, and, where available, region/source characteristics, to better document dataset comparability and improve transparency. Accordingly, the reported benchmark results should be interpreted as performance under a controlled and widely used dataset design rather than as a bias-free representation of all true biological non-interactions.

Together, these positive and negative sets form the WT CLASH dataset used in this study for model development and internal evaluation.

#### CSR-1 CLASH dataset

For independent evaluation, we used the CSR-1 CLASH dataset introduced by Yang et al.^32^, which was also derived from the PRG-1 CLASH experiments of Shen et al.^45^ but constructed to provide an external test setting distinct from the WT CLASH training data. As in the WT dataset, chimeric reads were separated into piRNA and mRNA segments, the piRNA sequence was matched to annotated piRNAs, and the mapped mRNA region was extended to account for possible truncation during library preparation.

A stringent filtering strategy was again applied to define the positive set, ensuring that only high-confidence piRNA–mRNA interactions were retained. Negative samples were generated using the same general design principle as in WT CLASH: candidate non-interacting pairs were selected from outside the loose binding set (LBS), overlapping positives were excluded, and only energetically plausible candidates were retained. This yielded an independent benchmark containing both positive and negative examples under a construction protocol consistent with the WT dataset.

As with the WT CLASH negatives, these CSR-1 CLASH negative samples should be understood as challenging non-binding candidates rather than arbitrary random pairs. This design improves biological relevance and makes the classification task more stringent, but it may also introduce some degree of dataset-specific bias because the negatives are defined through outside-LBS selection and energy-based filtering.

Thus, the CSR-1 dataset serves as a controlled external benchmark for assessing model generalization, while still sharing the same practical limitations inherent to this widely used negative sampling strategy.

#### Basic sequence properties in the positive and negative datasets

Table 2 compares the basic sequence properties of the positive and negative sets in WT CLASH and CSR-1 CLASH. The results show that the two classes are exactly matched in mean piRNA length and mean target-site length across both datasets, indicating that the classification task is not trivially driven by sequence length differences. The mean piRNA GC content is also highly similar between classes, especially in WT CLASH, where the positive and negative sets are identical in this respect. The main remaining difference is observed in target-site GC content, which is moderately higher in the positive samples than in the negative samples in both datasets. Thus, the table suggests that the benchmark is reasonably balanced in its basic structural properties, although some residual compositional bias may remain, particularly at the target-site level. Overall, these statistics support the use of the dataset as a challenging and biologically plausible benchmark, while also motivating caution in interpreting results as completely free from sampling bias.

**Table 2:** Descriptive comparison of positive and negative sample distributions in the WT CLASH and CSR-1 CLASH datasets.

Dataset	Class	n	Mean piRNA length	Mean target-site length	Mean piRNA GC	Mean target-site GC
WT CLASH	Positive	60,438	21.0	31.0	0.3968	0.4721
WT CLASH	Negative	60,438	21.0	31.0	0.3968	0.4287
CSR-1 CLASH	Positive	10,000	21.0	31.0	0.3837	0.4574
CSR-1 CLASH	Negative	10,000	21.0	31.0	0.3942	0.4273

Because the benchmark data provide fixed 31-nt target-site windows, we evaluated only shorter effective lengths via truncation, rather than testing larger values that would introduce only padding. The final model uses the full 31-nt target window, which yielded the best performance in our sensitivity analysis.

In the current benchmark, inputs are effectively fixed at 21 nt for piRNA and 31 nt for mRNA, so padding effects are minimal in the final experiments. Padded one-hot positions contribute zeros to the Pair-ID and compatibility channels, while the positional channel Δ is computed over the fixed interaction grid. Under more variable-length settings, explicit masking would be preferable to prevent padded positions from influencing Δ-related computations.

### The overall deep learning model

**Fig. 1:**
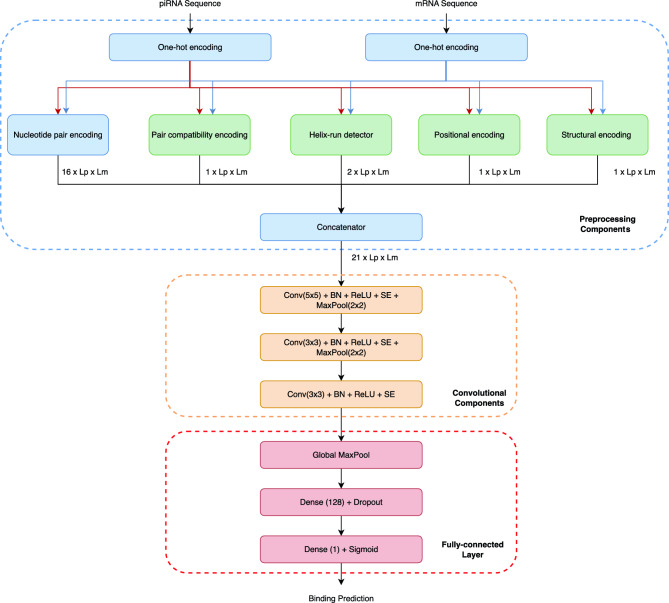
Overall AI Model. Data processing from RNA sequence inputs to final binding prediction is illustrated. Preprocessing is colored with blue and green. The green-colored components add physical data to the classical nucleotide-pair data. The orange components are used to extract convolutional interactions. Red components transform the data coming from convolution layers to the final binding prediction.

The end-to-end data flow of the proposed deep learning model is illustrated in Fig. 1. Blue and green components are responsible for data preprocessing. The two RNA sequences are first passed through a one-hot encoder. This is a widely used technique to represent different input types with equal numerical weights.

Four components build representations from the one-hot encoded input sequences. The details of these representations are explained in the next section. Here, we emphasize that the green-colored components add novelty to the presented AI model. Nucleotide pair encoding is a common technique for representing 16 different nucleotide pairs: AA, AC, AG, AT, CA, CC, CG, CT, GA, GC, GG, GT, TA, TC, TG, TT. We introduce some important physical knowledge to the input using the green components. The green components build 5 additional channels (1 compatibility, 2 helix-run, 1 positional, 1 structural), which are concatenated with the classical 16 nucleotide-pair channels, resulting in 21 channels in total.

After preprocessing, 3 convolutional layers are used to detect local patterns meaningful for making interaction decisions. We have 5 sub-components in a convolutional group layer: convolution, batch normalization, ReLU, squeeze-and-excitation, and MaxPool (orange in Fig. 1).

The convolutional layer helps identify local patterns in how piRNA and mRNA interact^46^. Analyzing interaction sequences^46^ and employing attention models^47^ supported learning in recent works. The local patterns in RNA sequences include important features such as seeds, mismatches, and regions where their structures complement each other. A 5 × 5 kernel size is selected to effectively capture short-range dependencies while giving the model flexibility to adapt across various pair positions within the interaction tensor. After the initial learning phase with larger kernels, the network shifts to fine-tuning its understanding by paying closer attention to finer details in later layers. By reducing the kernel size to 3 × 3 in convolutional layers 2 and 3, the model gets better at spotting even the tiniest, more delicate patterns in the input, all while keeping a broad view of the overall picture.

Batch normalization (BN) is a helpful technique that adjusts the output of the previous layer so that it has a mean of 0 and standard deviation of 1, across the batch. BN makes training more stable and faster. It also reduces the risk of vanishing or exploding gradients by keeping the data flowing through the network stable during training.

ReLU sub-component applies an activation function. ReLU function generates a zero output for all negative inputs, and it passes all positive inputs directly without any change. Zero input generates a zero output. ReLU adds nonlinearity to the model, making it easier to capture more complex patterns, since linear models can only learn simple, straight-line relationships. Plus, it promotes quicker training because it doesn’t suffer from saturation as some other activation functions do.

Squeeze-and-excitation (SE) is a helpful channel attention mechanism that makes the model more attentive to the most important features. It works by gathering global information about each channel, similar to understanding which features matter most for the task at hand—and then reweights these channels accordingly. The process starts with a squeeze step, where a global average pooling gives a summary for each feature map, highlighting, for example, which base-pair types are more informative. Then, in the excitation step, this information is passed through two fully connected layers with a ReLU activation in between, generating a set of weights between 0 and 1 for each channel. These weights help scale the feature maps, emphasizing the most key channels and attenuating less informative channels. In this way, SE helps the model focus on the most meaningful base-pair types, compatibility features, or regions, making its predictions more accurate and reliable.

Max pooling is a simple and effective down-sampling method. In MaxPool (2 × 2), a small 2 × 2 window scans the input tensor, picking out the highest value in each area. This process helps reduce the size of the input data, making the model more robust by reducing its sensitivity to the precise positions of features. As a result, the network can better recognize overall patterns rather than getting overly sensitive to local positional variation.

After the convolutional groups, the representation is progressively expanded to 256 feature maps (in the final convolutional block), which are then aggregated via global pooling and passed to the fully connected layers. The global MaxPool layer at the start of the red-colored fully connected layers produces a single final feature value for each channel. The first dense layer reduces the feature dimensionality to 128, and a second dense layer generates the final representation size. This number is then passed through a sigmoid function to produce the final prediction.

### The preprocessing components

**Fig. 2:**
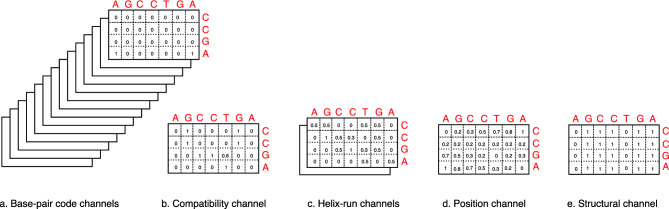
Classical base-pair channels and biophysics-informed data channels for an imaginary example RNA pair “CCGA” and “AGCCTGA”. In addition to the classical base-pair code channels, one compatibility channel, two helix-run channels, one position channel, and one structural channel are added. The numbers are rounded to only 1 decimal digit.

The novelty of the proposed model lies in the additional preprocessing components. Five channel groups are concatenated before being passed to the computational layer. Figure 2 displays these five data channel groups (16 base-pair channels + 5 additional channels). An imaginary piRNA sequence “CCGA” and an imaginary mRNA sequence “AGCTGA” are used in the figure. The preprocessing steps are given in Algorithm 1.

#### Biophysics-informed interaction tensor

The proposed input representation is a multi-channel interaction tensor constructed by combining sequence-level pairing information with biophysics-inspired priors. The tensor includes:

- base-pair identity channels (16 combinations),
- compatibility prior channel,
- helix-run related channels,
- positional encoding,
- structural accessibility signal.

While several of these components individually reflect known biochemical or structural intuition, their unified tensorized formulation for direct CNN-based learning of piRNA–mRNA interactions is, to our knowledge, novel in this task setting.

#### Base-pair code channels

Figure 2a shows the classical base pair representation where each of the 16 possible pairs is one-hot encoded. The first matrix is for the A-A pair; since there are 2 A-A pairs in this example, the corresponding entries are 1, and the remaining entries are 0. The base interaction code for the A-A pair is 1000000000000000, so the first matrix in Fig. 2a has a value of 1 at the positions corresponding to this pair. All other matrices have a value of 0 at this position. The following subsections explain the additional channels that make the model biophysics-informed.

#### Compatibility channel

We add a channel that informs the convolution layers about the well-known binding properties of incoming nucleotide pairs. A constant 4 × 4 S matrix is created to represent Watson–Crick pairs “A-U, U-A, G-C, and C-G” with 1, wobble pairs “G-U and U-G” with 0.6, and other pairs with 0^48^. This matrix thus encodes the biophysics-informed binding possibilities. We follow the encoding order A = 0, C = 1, G = 2, and U = 3, and the resulting S matrix is defined as shown in Eq. 4.

Using this S matrix, we generate a complementarity map for the piRNA and mRNA sequences in question. A complete mathematical model will be detailed in the following sections, so here we focus on introductory data. We first multiply the P matrix, which contains the one-hot encoded piRNA sequence, with S. Then, the resulting matrix is multiplied by M^T^, the transpose of the M matrix that contains the one-hot encoded mRNA sequence. Then a clamping function restricts the results between 0 and 1. Considering the example pair, the resulting complementarity map is given in Fig. 2b. Algorithm 1, Step 2, gives the operations needed to build the one-channel complementarity matrix.

#### Helix-run channels

Another group of channels is added to inform the CNN layers about the length of continuous Watson–Crick and Wobble pairs in the two RNA sequences. We built 2 channels, one for diagonal matches, and another one for anti-diagonal matches.

Algorithm 1, Step 3, shows how these two Helix-run channels are built. First, we choose a k value as the size of the convolution kernel. For our imaginary small sequences in Fig. 2, we use k = 2. K_diag_ is the convolution kernel, which is an identity matrix of size k x k, and K_anti_ is used as a second kernel to detect reverse continuous possible binding sites and is obtained by taking the horizontal flip of K_diag_. Then we convolve these kernels with the C compatibility matrix obtained in Step 2 and divide the result by k to restrict the helix-run results between 0 and 1. Resulting H_diag_ and H_anti_ matrices record helix-run data about our RNA sequence pairs.

#### Positional channel

The relative position of the base pairs in their RNA sequences is another important data that may affect the binding interaction. So, we build one more channel that will hold the relative position information of the pairs.

Algorithm 1 – Step 4 shows how to obtain the matrix containing relative position data. First, we build an I matrix that records the positions of nucleotides in the piRNA sequence as floating-point numbers between 0 and 1. The position numbers are reflected in different rows, and all the numbers in a specific row are identical. Similarly, we build another matrix, J, which holds the position information of the bases in the mRNA sequence. Inside each column in J, all rows have identical numbers, and the position data is reflected in different columns. Then, by taking the absolute differences of corresponding elements in the I and J matrices, Δ is built to record the relative positions of possibly interacting base pairs.

#### Structural channel

The structure of the RNA sequences is very important, because some nucleotides may be paired already within the RNA molecule due to its structure and they may not be available for a binding to a secondary RNA.

The Nussinov algorithm^49^ is an old but useful method to predict the pairing status of the RNA nucleotides. It attempts to find the most stable structure (maximizing the number of base pairs) according to canonical rules (A–U, G–C, and sometimes G–U). A recursion fills a DP matrix with the best pairing score for subsequences. Ultimately, a traceback identifies which nucleotides are paired and which remain unpaired. For our case, we built two vectors, up and um, for piRNA and mRNA sequences. A 0 in these vectors indicates that the nucleotide is predicted to be paired already and not available for binding to outer molecules. On the other hand, a 1 indicates that the related base is unpaired. We build the structural channel matrix A by taking the outer product of these two vectors.

The structural channel is designed as an accessibility prior rather than a free-energy descriptor. Accordingly, we use Nussinov folding to obtain a paired/unpaired mask that approximates whether each nucleotide is likely to be structurally constrained. We emphasize that this mask is used as an auxiliary feature and is not treated as ground-truth secondary structure. Nussinov was chosen for its computational efficiency and deterministic behavior, which enables scalable preprocessing over large datasets. Thermodynamics-based alternatives (e.g., ViennaRNA/RNAstructure, pairing probabilities, or unpairedness measures) could be substituted within the same channel definition and are considered in future work.

In our model, for each sequence, an approximate folding is computed using a Nussinov-style dynamic programming algorithm, and nucleotides are labeled as paired or unpaired. This information is encoded as an unpairedness mask, where unpaired positions are assigned higher accessibility.

For a given piRNA–mRNA pair, the structural channel is constructed as the outer product of the two unpairedness vectors, resulting in a matrix that reflects joint accessibility of interacting positions.

This feature is used as a biophysically motivated prior to guide learning and should not be interpreted as ground-truth structural annotation.

#### Concatenation

Final operation in preprocessing is combining the matrices obtained in the initial 5 steps and building a tensor of the shape 21 × Length_piRNA_ x Length_mRNA_. 16 channels come from base-pair interaction encoding, 1 channel is for compatibility data, 2 channels hold forward and backward helix-run information, 1 channel holds relative position information of the base pairs, and 1 channel records structural pairing information.

### Mathematical model

The proposed model needs an alphabet of valid nucleotides.

Let *Z* = {A, C, G, U} be the alphabet for RNA sequences. Please note that all T bases are mapped to U in the preprocessing step.

If l_p_ is the length of a piRNA sequence, then a piRNA sequence can be written as p = (p_0_, p_1_, . . . , p_lp-1_) ∈ *Z*^lp^ . Similarly, we can write an mRNA site sequence as m = (m_0_, m_1_, . . . , m_lm-1_) ∈ *Z*^lm^ .

Now, let Lp and Lm be the maximum allowed lengths of a piRNA and mRNA, respectively. We either right-pad or truncate the incoming strings to ensure that lp ≤ Lp and lm ≤ Lm. Therefore, within the model, the lengths used are always Lp and Lm.

We apply one-hot encoding and generate the P and M matrices as follows:

1 $$\begin{array}{c}P :=H\left(p\right)\in {\left\{\mathrm{0,1}\right\}}^{{L}_{p}\times 4}\end{array}$$

2 $$\begin{array}{c}M :=H\left(m\right)\in {\left\{\mathrm{0,1}\right\}}^{{L}_{m}\times 4}\end{array}$$

**Algorithm 1:**
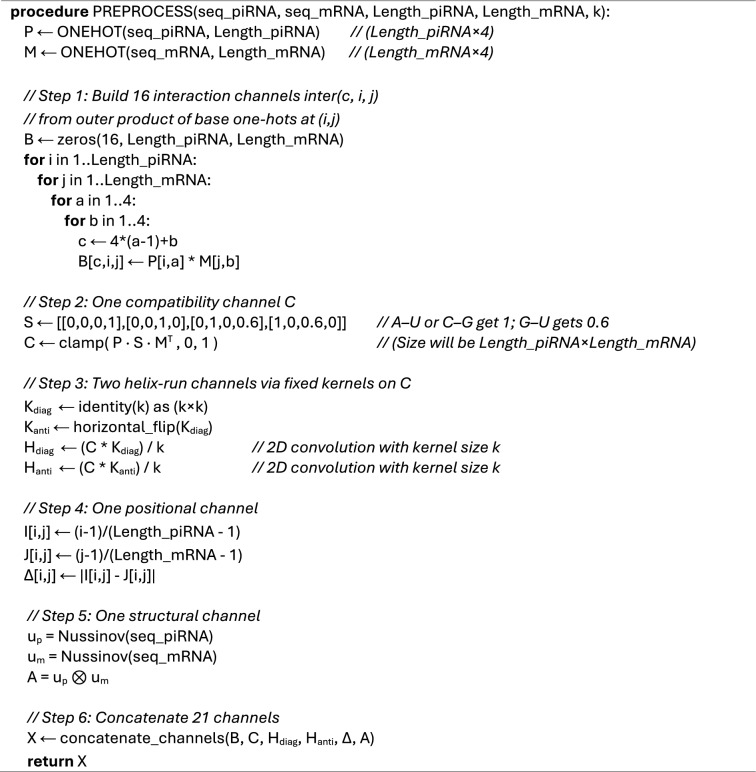
Preprocessing of the input RNA sequences.

The input to the CNN layers is a tensor X ∈ ${\mathbb{R}}$ ^Ch x Lp x Lm^ where Ch = 21 channels. These 21 channels are concatenated from 16 pair-identity interaction channels, 1 compatibility prior channel, 2 helix-run channels, 1 positional channel, and 1 structural channel.

To get the 16 pair-identity channels, we take the outer product of one-hot encoded sequences represented as p and m. Let P_i,a_ be the one-hot encoding for a piRNA sequence p and M_j,b_ for an mRNA sequence m, where a, b ∈ {A, C, G, U} indexed as {0,1,2,3}. For each nucleotide pair (a,b), we define the pair-identity matrix B_a,b_ as below.

3 $$\begin{array}{*{20}c} {B_{{a,b}} \left[ {i,j} \right]: = P_{{i,a}} \cdot M_{{j,b}} \in \left\{ {0,1} \right\}} \\ \end{array}$$

Stacking the 16 combinations results in a tensor B ∈ {0,1}^16 x Lp x Lm^.

Then, using the compatibility matrix in Eq. 4 that encodes the Watson–Crick and wobble pairs, we build the compatibility channel as defined below.

4 $$\begin{array}{c}S :=\left[\begin{array}{cccc}0& 0& 0& 1\\ 0& 0& 1& 0\\ 0& 1& 0& 0.6\\ 1& 0& 0.6& 0\end{array}\right]\end{array}$$

5 $$\begin{array}{c}C={f}_{clip\left[\mathrm{0,1}\right]}(P\bullet S\bullet {M}^{T}) \in {[\mathrm{0,1}]}^{1\times {{L}_{p}\times L}_{m}}\end{array}$$

In Eq. 5, the results are mapped to the range [0,1] using a clipping function.

To detect local diagonal runs (indicative of helices), we apply 2D convolutions to the compatibility channel using small k × k kernels that capture diagonal and anti-diagonal runs. We use a diagonal kernel K_diag_​, which is an identity matrix of size k, so it responds to alignments (i + t, j + t). Besides, we use an anti-diagonal kernel K_anti_ to capture anti-diagonal alignments (i + t, j-t). Formally, we can express these operations as in Eqs. 6 and 7.

6 $$\begin{array}{c}{H}_{diag}=\frac{1}{k}Conv2D(C,{K}_{diag}) \in {\mathbb{R}}^{1\times {{L}_{p}\times L}_{m}}\end{array}$$

7 $$\begin{array}{c}{H}_{anti}=\frac{1}{k}Conv2D(C,{K}_{anti}) \in {\mathbb{R}}^{1\times {{L}_{p}\times L}_{m}}\end{array}$$

Positions are encoded by normalizing the location of each nucleotide as in Eq. 8.

8 $$\begin{array}{c}{i}{\prime} = \frac{i}{{L}_{p}-1} , {j}{\prime} = \frac{j}{{L}_{m}-1}\end{array}$$

Now we can define the positional delta channel as in Eq. 9.

9 $$\begin{array}{c}\Delta \left[i,j\right]= \mid {i}{\prime}-{j}{\prime}\mid , \Delta \in {\mathbb{R}}^{1\times {{L}_{p}\times L}_{m}}\end{array}$$

For each sequence, we compute an unpairedness indicator vector u(s) ∈ {0,1}^L^ using a Nussinov maximum-pair DP with minimum loop length l_min_ (we take l_min_ = 3). The Nussinov algorithm sets u(s)_i_ = 0 if the position i is predicted to be paired already and unavailable for new bindings and it is set to 1 if the position is predicted to be unpaired. This way, we build u_p_ ∈ {0,1}^Lp^ and u_m_ ∈ {0,1}^Lm^ vectors. The structure channel A is calculated as the outer product of these two vectors

10 $$\begin{array}{c}A\left[i,j\right]= {u}_{p}[i]\bullet {u}_{m}[j], A \in {\{\mathrm{0,1}\}}^{1\times {{L}_{p}\times L}_{m}}\end{array}$$

We concatenate all these channels to get the final tensor input to the convolution layers.

11 $$\begin{array}{c}X= concat(B,C, {H}_{diag}, {H}_{anti},\Delta ,A), X \in {\mathbb{R}}^{21\times {{L}_{p}\times L}_{m}}\end{array}$$

Using this X tensor, CNN layers and fully connected layers produce a prediction value, and the final prediction is done by means of a sigmoid function.

### Algorithmic complexity of added preprocessing operations

It is known that the complexity of Nussinov algorithm is $\mathcal{O}({n}^{3})$. However, we assume that the Nussinov pairedness data is given together with the RNA sequence, and it is not included in complexity. If it is not available, the code we use calculates the pairing predictions and saves them in a file to use in future occasions.

In Step 1 of Algorithm 1, there are 4 nested for loops, but 2 of them run four times constantly, so they do not increase complexity. So, the complexity of Step 1 is $\mathcal{O}({{L}_{p}\bullet L}_{m}$).

In Step 2 the operation P · S · M^T^ runs on P matrix of size L_p_ × 4 and M matrix of size L_m_ × 4. The size of the S matrix is constantly 4 × 4. So, the complexity of Step 2 is also $\mathcal{O}({{L}_{p}\bullet L}_{m}$).

In Step 3, a fixed size convolution matrix is scanning all the elements of matrix C of size L_p_ x L_m_. Since a constant number of convolution operations are handled L_p_ x L_m_ times, the complexity is $\mathcal{O}({{L}_{p}\bullet L}_{m}$).

In Step 4, we need $\mathcal{O}({{L}_{p}\bullet L}_{m}$) operations to determine the relative positions of possibly interacting base-pairs.

So, if channel number is Ch, maximum sequence lengths are L_p_ and L_m,_ and structural pairedness information is provided by the dataset, the complexity of Algorithm 1 is $\mathcal{O}(Ch\bullet {{L}_{p}\bullet L}_{m}$), where the number of channels is 21 and constant. So, the final complexity for the preprocessing operations for ready Nussinov structural predictions is $\mathcal{O}({{L}_{p}\bullet L}_{m}$). We can conclude that if ready Nussinov structural predictions are provided, the added preprocessing operations do not increase complexity.

### Network configuration and training protocol

#### Network configuration

The final rbpCNN model uses a 21-channel input tensor and three convolutional blocks. Block 1 applies a 2D convolution with 64 channels, kernel size 5 × 5, stride 1, and padding 2, followed by batch normalization, ReLU, squeeze-and-excitation (SE), and 2 × 2 max pooling. Block 2 uses 128 channels with kernel size 3 × 3, stride 1, and padding 1, again followed by batch normalization, ReLU, SE, and 2 × 2 max pooling. Block 3 uses 256 channels with kernel size 3 × 3, stride 1, and padding 1, followed by batch normalization, ReLU, and SE. The SE blocks use a reduction ratio of 8. After the convolutional blocks, global max pooling is applied, followed by a fully connected layer 256 → 128, ReLU, dropout (0.50), and a final linear layer 128 → 1 for logit prediction.

#### Training protocol

The model was trained using Adam with learning rate 1 × 10⁻^3^, batch size 64, weight decay 1 × 10⁻^4^, and dropout 0.50. Early stopping was applied based on validation ROC-AUC with patience 4 epochs. A ReduceLROnPlateau scheduler was used with factor 0.5 and patience 2. All experiments used random seed 42 for Python, NumPy, and PyTorch.

#### Input lengths and padding/truncation

Let L_p_ and L_m_ denote the fixed piRNA and mRNA-site lengths used by the network. After cleaning the sequences, inputs were right-padded or truncated to these lengths before one-hot encoding and tensor construction. In the implementation, L_p_ and L_m_ are set to the maximum cleaned lengths observed in the dataset used for training.

### Helix-run kernel

Unless otherwise stated, the helix-run channels use kernel size k = 7, with same-padding ⌊k/2⌋ and normalization by k. A sensitivity analysis over k ∈ {5, 7, 9} is reported in Table 5 and indicates that performance is stable across this range, with k = 7 used in the final experiments.

#### Threshold selection

Within each fold, a decision threshold was selected on the inner validation split by sweeping thresholds and maximizing Youden’s J statistic. That fold-specific threshold was then used for the held-out fold evaluation. For the fully independent CSR-1 dataset, a fixed threshold of 0.5 was used to avoid post hoc tuning on the external test set.

### Experimental setup and assisting tools

We used Python Language and PyTorch open-source deep learning library to implement possible solution algorithms. The codes are run on a Windows PC having the following specs: 13^th^ Gen Intel Core i7-13700H CPU, NVIDIA GeForce RTX 4060 Laptop GPU, 32 GB RAM, 1 TB Hard Drive.

## Results

To better understand how the additional channels enhanced prediction efficiency, we presented sample- based and dataset-based Grad-CAM images from both the basic 16-channel CNN model and our 21-channel rbpCNN model.

We also created per-channel average saliency plots for the proposed model, obtained from the entire independent test dataset, to highlight the unique contribution of each added channel to the prediction process. The final subsection shares the AI model’s accuracy and AUC performance results.

### Grad-CAM comparison for sample pairs

**Fig. 3:**
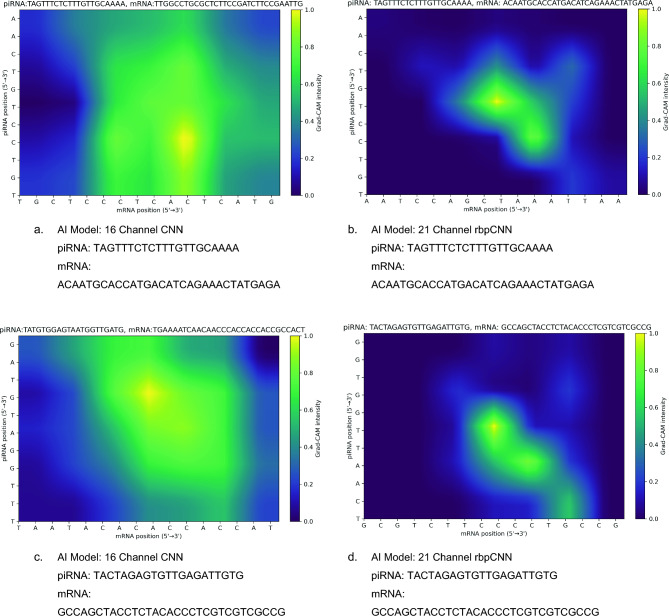
Grad-CAM heatmap images obtained by 16 channel CNN model and 21 channel rbpCNN model for randomly selected 2 **positive** pairs. The same piRNA and mRNA sequences are used in a row. Left image shows the Grad-CAM heatmap for 16 channel model and the right image in a raw depicts the Grad-CAM heatmap obtained by the proposed rbpCNN model.

To understand how the extra five structural and compatibility channels influenced the model’s interpretability and binding predictions, we examined Grad-CAM heatmaps for the same positive piRNA–mRNA pairs shown in Fig. 3.

Grad-CAM images help us understand how a trained CNN makes decisions by tracing backward from its predictions. First, we pass the input piRNA–mRNA pair through the network to get a binding prediction. Then, we calculate gradients of this prediction with respect to the last convolutional feature maps to see which features had the strongest influence. These gradients are averaged to give an importance weight to each feature map. The maps are combined into a heatmap that highlights the most relevant areas. After applying a ReLU to keep only positive influences and resizing the heatmap to match the input size, we visualize it as a color map. The bright regions show where the model focused most to make its decision.

In both examples shown in Fig. 3, the baseline 16-channel CNN (left panels, Fig. 3a and c) tends to produce more widespread activation patterns, covering larger areas of moderate intensity across the sequence alignment. This suggests that the baseline network tends to spread importance across many nucleotide positions, indicating less precise identification of the key binding sites.

On the other hand, the 21-channel proposed model (right panels, Fig. 3b and d) displays more focused and sharper activation regions. In both positive pairs, the Grad-CAM highlights clear stretches of consecutive nucleotides that match known piRNA–mRNA binding regions. The more concise and targeted activation implies that incorporating structural and contextual features—such as compatibility, helix-run, positional difference, and structural channels—helps the network better capture biologically meaningful interaction signals, rather than relying on diffuse, pairwise features alone.

**Fig. 4:**
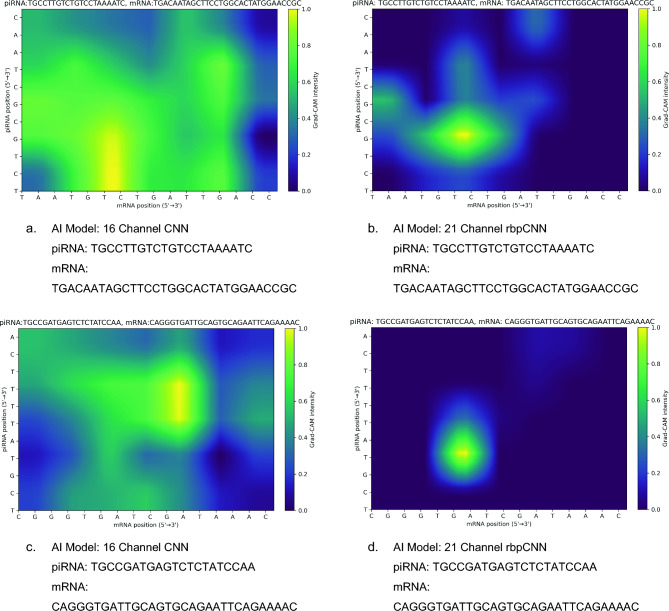
Grad-CAM heatmap images obtained by 16 channel CNN model (left column) and 21 channel rbpCNN model (right column) for randomly selected 2 **negative** pairs. The same piRNA and mRNA sequences are used in a row.

Looking at the Grad-CAM results for negative pairs in Fig. 4, we notice a distinct difference between the baseline 16-channel CNN and the proposed 21-channel model.

For the 16-channel CNN (left panels), the heatmaps show broader and more diffuse activation zones across the sequence, indicating that the model is less decisive in identifying non-binding interactions. It highlights multiple positions with moderate intensity, even when the pairs are known to be negative. These widespread attention patterns might reflect uncertainty or a reliance on less discriminative features.

In contrast, the 21-channel model (right panels) produces much sharper and localized Grad-CAM activations. The intensity is concentrated in a few narrow regions, while most of the sequence space remains at very low activation. This indicates that the enriched feature set (compatibility, helix-run, positional difference, and structural channels) enables the model to more confidently rule out spurious binding by focusing only on limited positions of partial complementarity.

These results indicate that the inclusion of the five additional channels improves the network’s ability to make binding predictions with higher confidence and stronger localization for both positive and negative pairs. This visual evidence shows that the proposed model attributes binding to narrower, biologically interpretable regions of the piRNA–mRNA duplex.

### Average Grad-CAM comparison for the whole independent dataset

**Fig. 5:**
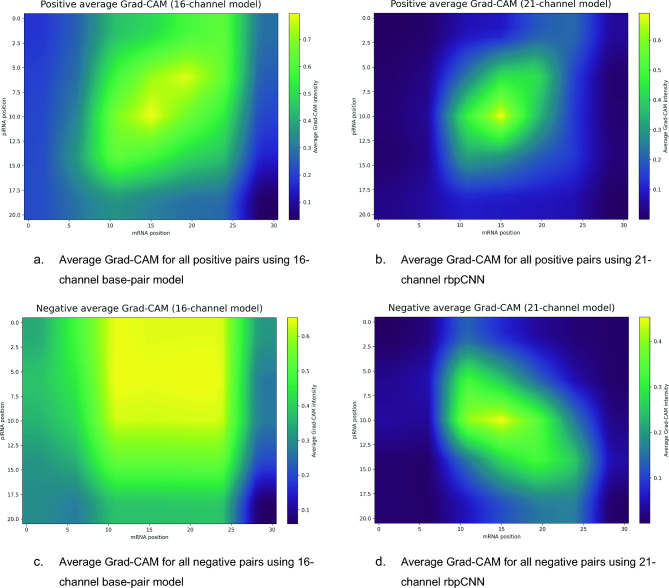
Average Grad-CAM heatmap images obtained by 16 channel CNN model (left column) and 21 channel rbpCNN model (right column) for all positive and negative pairs in the independent test dataset.

To move beyond anecdotal example-based interpretation, we computed average Grad-CAM heatmaps over all positive and all negative pairs in the independent CSR-1 dataset for both the 16-channel base-pair model and the 21-channel rbpCNN. This dataset-level analysis provides a more stable view of which interaction regions each model consistently emphasizes. The average Grad-CAM images are given in Fig. 5.

For the positive pairs, both models assign elevated importance to a central interaction zone, indicating that prediction is driven by localized regions rather than uniformly distributed evidence across the entire tensor. However, the two models differ substantially in how sharply this importance is concentrated. The 16-channel model produces a broader and more diffuse activation pattern extending over a relatively large portion of the interaction map, suggesting that it relies on a wider but less selective region of pairwise evidence. In contrast, the 21-channel rbpCNN yields a much more focused hotspot, indicating that the addition of biophysics-informed channels encourages the model to concentrate on a more specific and coherent interaction region. This sharper localization is consistent with the idea that the added priors help suppress irrelevant background structure and guide attention toward more biologically meaningful interaction patterns.

For the negative pairs, the distinction between the models is even more informative. The 16-channel model still shows relatively strong and spatially broad activation over a large central region, implying that it continues to detect substantial pairwise signal even in non-interacting examples. This may reflect reduced specificity, where local matching patterns alone can still trigger high internal responses. By contrast, the 21-channel rbpCNN exhibits a weaker, more structured, and more spatially confined response in the negative set. Although some activation remains, it is less uniformly spread and appears more discriminative than in the 16-channel model. This suggests that the additional channels improve the model’s ability to distinguish biologically plausible interactions from spurious pairwise similarity.

Taken together, the averaged Grad-CAM maps support two main conclusions. First, both models learn non-random, spatially organized interaction patterns, confirming that the learned decision process is structured rather than arbitrary. Second, the 21-channel rbpCNN produces more concentrated and better differentiated saliency patterns between positive and negative samples, indicating improved interpretability and specificity relative to the 16-channel baseline. In this sense, the biophysics-informed channels do not merely increase input dimensionality; they appear to refine where the model looks, making its decisions more localized and more consistent with a selective interaction mechanism.

Overall, the whole-dataset average Grad-CAM analysis strengthens the interpretability claims of the model by showing that the observed saliency structure is not limited to a few handpicked examples, but is instead reproducible across the independent test set as a whole.

### Per-channel saliency results

Per-channel saliency analysis was used to quantify the extent to which the model relies on different feature groups across the entire independent test set. For each piRNA–mRNA pair, the model first produces a logit through a forward pass. We then compute the gradient of this logit with respect to the input tensor, obtaining a saliency map across all channels and interaction positions. These gradients are aggregated within each channel and then grouped into biologically meaningful feature categories: Pair-IDs (16 base-pair identity channels), compatibility (C), helix-run features (Runs: diagonal + anti-diagonal), positional difference (Δ), and structural accessibility (A). Finally, the grouped contributions are normalized and averaged over all samples in each subset to reveal which feature groups systematically influence the model’s predictions.

**Fig. 6:**
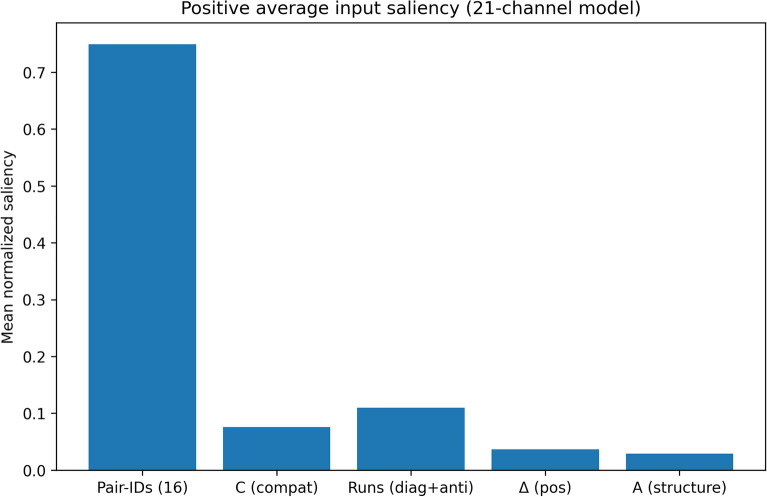
Average per-channel saliency distribution for the independent positive test set.

**Fig. 7:**
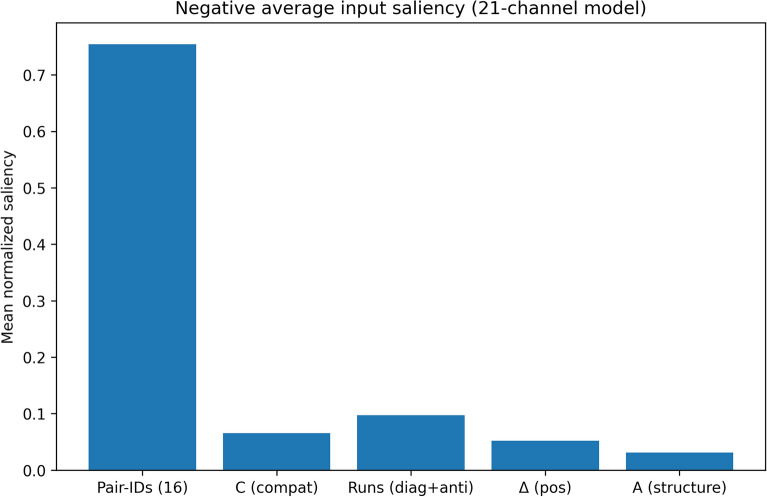
Average per-channel saliency distribution for the independent negative test set.

Table 3 summarizes the average per-channel saliency values for the independent positive and negative test sets, and Figs. 6 and 7 visualize these average group contributions.

**Table 3:** Average per-channel saliency results for independent positive and negative test sets.

Subset	Mode	n_samples	Mean Pair-IDs (16)	Mean C (compat)	Mean Runs (diag + anti)	Mean Δ (pos)	Mean A (structure)
Positive	21	10000	0.749447	0.075727	0.10951	0.036404	0.028912
Negative	21	10000	0.754387	0.065428	0.097083	0.052212	0.03089

The results show that the model is dominated by the Pair-IDs (16) group in both subsets. For the positive set, Pair-IDs account for 0.749 of the normalized saliency, while for the negative set they account for 0.754, indicating that direct nucleotide pairing patterns form the principal basis of the model’s decision-making in both interacting and non-interacting pairs. This confirms that the network primarily learns from the core pairwise interaction representation.

Among the auxiliary feature groups, the helix-run features provide the second strongest contribution, with an average saliency of 0.110 in the positive set and 0.097 in the negative set. This suggests that local run-like interaction patterns are informative and are used more strongly in predicted positive interactions than in negatives. The compatibility channel (C) also contributes meaningfully, with average saliency values of 0.076 for positives and 0.065 for negatives, again showing a somewhat stronger role in the positive subset.

In contrast, the positional difference channel (Δ) and the structural accessibility channel (A) make smaller overall contributions. For positive samples, Δ contributes 0.036 and A contributes 0.029. For negative samples, Δ increases to 0.052, while A remains low at 0.031. The larger Δ contribution in the negative subset may indicate that positional discrepancy is used more as a weak corrective or discriminative cue when the model evaluates non-binding patterns, rather than as a primary determinant of positive interaction.

Overall, these whole-dataset averages show that the model relies most strongly on direct base-pair identity information, while the additional biophysics-informed channels provide secondary but non-negligible support. Importantly, these auxiliary channels do not replace the main pairwise interaction signal; instead, they refine the decision process by adding complementary information. In the positive set, the non-Pair-ID channels together contribute about 25.1% of the total saliency, while in the negative set they contribute about 24.6%, demonstrating that the added priors consistently participate in the model’s reasoning across the full independent dataset.

These findings support the interpretation that rbpCNN is fundamentally driven by biologically meaningful pairing patterns, with compatibility and helix-run signals providing the most useful complementary information, while positional and structural priors play a smaller, more context-dependent role.

### Ablation study on full training and independent datasets

To systematically evaluate the contribution of each input component, we conducted two complementary ablation analyses: (i) leave-one-channel-out from the full model (Table 4), and (ii) single-channel-only models (Table 5). All experiments were performed on the WT training split and evaluated on both WT and the independent CSR-1 dataset.

**Table 4:** Ablation results on removing each channel individually.

Model	WT ROC-AUC	WT PR-AUC	CSR-1 ROC-AUC	CSR-1 PR-AUC
Full model	0.9647	0.9704	0.9412	0.9502
Full—Base16	0.9459	0.9569	0.9168	0.9328
Full—C	0.9646	0.9706	0.9403	0.9506
Ful—H_diag_	0.9661	0.9719	0.9429	0.9522
Ful—H_anti_	0.9648	0.9706	0.9388	0.9489
Full—Δ	0.9654	0.9708	0.9385	0.9490
Full—A	0.9651	0.9711	0.9389	0.9493

**Table 5:** Ablation results on employing each channel alone.

Model	WT ROC-AUC	WT PR-AUC	CSR-1 ROC-AUC	CSR-1 PR-AUC
16-channel baseline	0.964247	0.969894	0.938865	0.948624
Only C	0.945333	0.955557	0.915879	0.931651
Only Hdiag	0.899606	0.914933	0.863056	0.881982
Only Hanti	0.933767	0.947893	0.900275	0.920598
Only Δ	0.5	0.5	0.5	0.5
Only A	0.536875	0.525073	0.532765	0.516891

Table 4 shows that removing the 16-channel pair-identity tensor (Full − Base16) leads to a substantial performance drop (WT ROC-AUC: 0.9647 → 0.9459; CSR-1 ROC-AUC: 0.9412 → 0.9168), confirming that base-pair interaction encoding forms the core predictive signal of the model.

In contrast, removing any of the additional biophysics-informed channels results in moderate changes, particularly on the independent CSR-1 dataset. Removing Hanti, Δ, or A yields noticeable decreases in CSR-1 ROC-AUC (~ 0.002–0.003) and PR-AUC. Removing C has minimal effect, suggesting that compatibility information is already partially captured within the pairwise encoding. Interestingly, removing Hdiag slightly improves performance, indicating some redundancy in that channel under the current configuration. Overall, these results show that no single auxiliary channel dominates performance. The gains of rbpCNN arise from the combined contribution of multiple complementary priors, particularly improving generalization to the independent dataset.

To further assess the intrinsic predictive power of each feature type, we trained models using each channel group in isolation. As depicted in Table 5, the 16-channel baseline alone already achieves strong performance (WT ROC-AUC ≈ 0.964), confirming that pairwise nucleotide interactions are the primary signal. The compatibility channel (C) alone retains moderate predictive power (WT ROC-AUC ≈ 0.945), indicating it captures meaningful biochemical constraints. The helix-run channels show asymmetric behavior: H_anti_ performs substantially better than H_diag_, suggesting anti-diagonal stacking patterns are more informative for piRNA targeting. The Δ (positional) and A (structural accessibility) channels alone perform near random, indicating they are not independently predictive, but rather act as contextual priors. These results demonstrate that auxiliary channels do not function as standalone predictors, but instead enhance performance when integrated with base interaction features. This supports the design principle of rbpCNN as a biophysics-informed augmentation, rather than a replacement, of sequence interaction modeling.

Ablation studies reveal that the 16-channel interaction tensor is essential for predictive performance. Additional channels provide incremental yet consistent improvements, especially for cross-dataset generalization. The improvements arise from complementarity rather than dominance, validating the multi-channel design.

### Deep learning model training performance

The proposed biophysics-informed rbpCNN performs consistently well across all five folds, as shown in Table 6, with an average AUC of 0.9655, indicating strong and stable performance on this benchmark. Other metrics, including accuracy (0.9066), precision (0.9242), recall (0.8860), and F1 score (0.9046), also indicate balanced and strong classification performance. Importantly, the standard deviations across folds are very small, emphasizing the model’s stability.

**Table 6:** Fivefold training and test results on WT_CLASH dataset.

Fold	Acc	Precision	Recall	F1	AUC	Threshold	Train time (s)	Test time (s)
1	0.9046	0.9147	0.8924	0.9034	0.9662	0.4500	97.9873	1.9483
2	0.9072	0.9184	0.8939	0.9060	0.9663	0.5050	95.0924	2.0826
3	0.9068	0.9273	0.8828	0.9045	0.9645	0.5150	88.1670	2.0419
4	0.9074	0.9411	0.8693	0.9038	0.9653	0.6450	112.1656	2.2048
5	0.9068	0.9194	0.8918	0.9054	0.9652	0.5250	83.1619	2.3060
Average	0.9066	0.9242	0.8860	0.9046	0.9655	0.5280	95.3148	2.1167

**Fig. 8:**
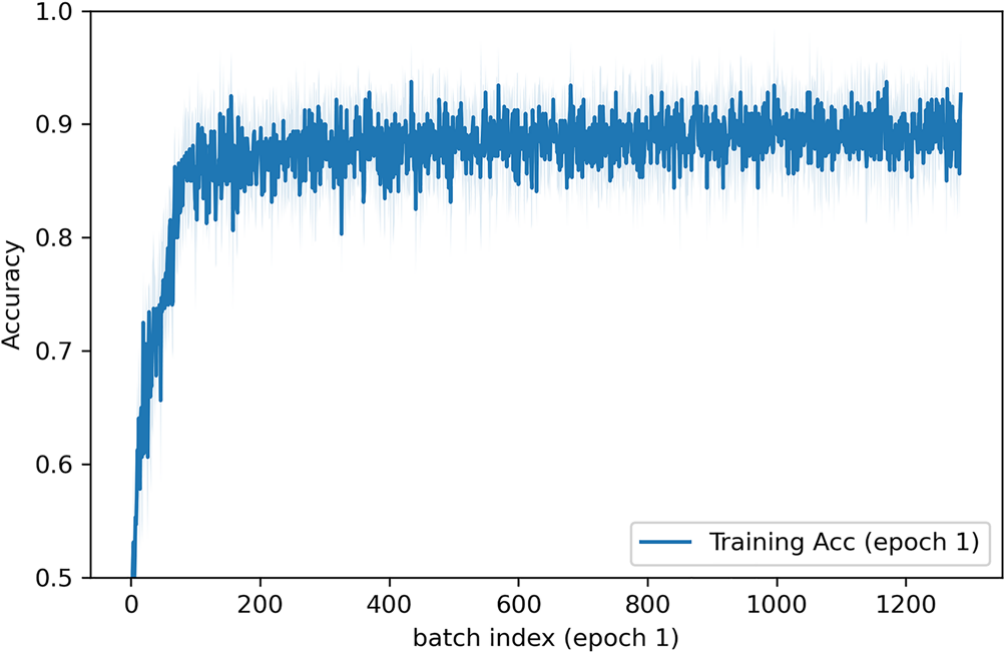
Accuracy changes as batches get processed in epoch 1.

**Fig. 9:**
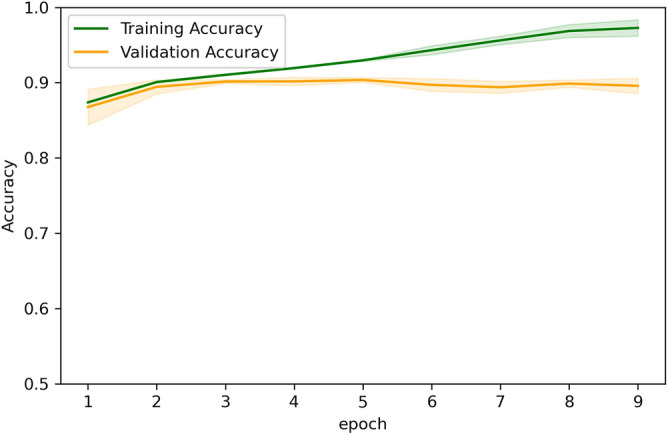
Training and validation accuracies vs epochs.

The training dynamics show that the model learns very quickly. As seen in Fig. 8, during the first epoch, the accuracy already nears its asymptotic value, indicating that the model captures most of the relevant discriminative information almost immediately. As shown in Fig. 9, by epoch 4, both the training and validation curves level off, with minimal additional improvement in later epochs. This suggests that training beyond 4 epochs offers little benefit, reducing the computational load.

In terms of efficiency, the training time per fold is brief (≈95 s on average), and inference is extremely quick (≈2.1 s on the test split). Such short runtimes make the method practical for large-scale screening applications where thousands of piRNA–mRNA pairs need to be evaluated.

**Fig. 10:**
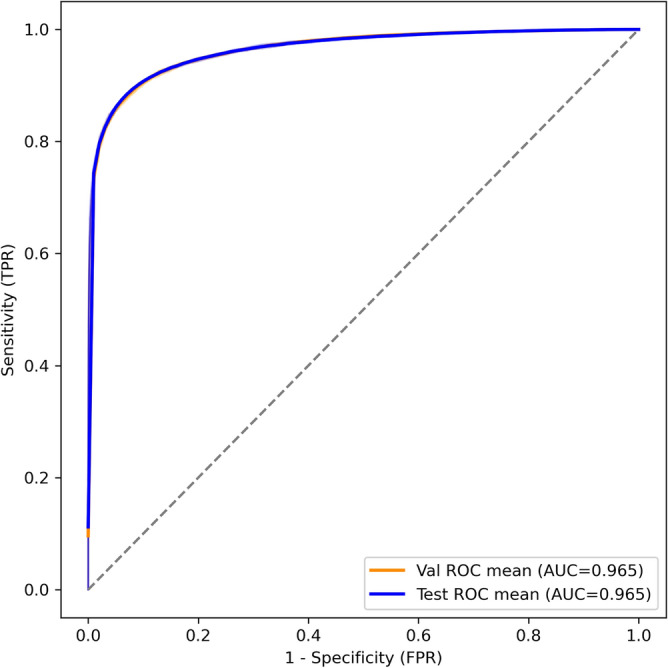
ROC curves for validation and test datasets on the WT CLASH dataset.

As shown in Fig. 10, the ROC curves for both the validation and test sets are nearly identical, with average AUC values of 0.965 for each. This suggests that the model generalizes extremely well, showing the same ability to distinguish between interacting and non-interacting piRNA–mRNA pairs on unseen data as it does during validation.

The sharp initial rise of the curves indicates that the model keeps a very high true positive rate even at low false positive rates, which is especially important in biological applications where false positives can be costly. The nearly identical performance on both validation and test sets also verifies that the regularization methods (dropout and weight decay) effectively prevent overfitting.

### Dependency on helix-run diagonal length

Helix-run channels are computed by applying fixed k × k convolutional filters along the diagonal and anti-diagonal directions of the compatibility matrix. Unless otherwise stated, we used k = 7, which is consistent with typical seed-region lengths in piRNA–mRNA interactions.

The diagonal filter captures contiguous matches along the main diagonal, while the anti-diagonal filter captures antiparallel alignment patterns corresponding to canonical base pairing. These two channels therefore encode complementary geometric interaction patterns.

To preserve spatial dimensions, the same padding of ⌊k/2⌋ is applied. The convolution outputs are normalized by k to ensure consistent scaling across different kernel sizes and to reduce boundary effects.

We evaluated the sensitivity of the helix-run kernel size k by testing k ∈ {5, 7, 9}. The results in Table 7 show that performance is stable across this range, with k = 7 yielding the best overall performance. This suggests that the model is not highly sensitive to the exact choice of k, as long as it remains within a biologically reasonable range.

**Table 7:** Effect of helix-run kernel size (k) obtained on the WT CLASH dataset.

k	ROC_AUC	PR_AUC	Accuracy	Precision	Recall	F1
5	0.965	0.9707	0.9074	0.9347	0.876	0.9044
7	0.9662	0.9713	0.9066	0.9461	0.8623	0.9022
9	0.9655	0.9712	0.9058	0.9114	0.8989	0.9051

The inclusion of both diagonal and anti-diagonal channels allows the model to capture both canonical antiparallel pairing and alternative local alignment patterns, improving robustness to positional shifts and mismatches.

### Sensitivity to mRNA length values

We evaluated the effect of truncating the target-site window by testing effective mRNA lengths of 21, 25, and 31 nt. As shown in Table 8, performance improved consistently as more target context was retained. On the WT set, ROC-AUC increased from 0.9495 at 21 nt to 0.9631 at 25 nt and 0.9662 at 31 nt, while PR-AUC increased from 0.9559 to 0.9688 and 0.9698, respectively. A similar trend was observed on the independent CSR-1 dataset, where ROC-AUC rose from 0.9228 to 0.9360 and 0.9419, and PR-AUC from 0.9334 to 0.9462 and 0.9469. These results indicate that shorter truncation removes useful binding context, whereas the full 31-nt window provides the best overall generalization. As expected, training cost increased with input length, from 140.1 s at 21 nt to 480.0 s at 31 nt, highlighting a trade-off between efficiency and predictive performance.

**Table 8:** Sensitivity to mRNA length (Lm).

Lm	WT_ROC_AUC	WT_PR_AUC	CSR1_ROC_AUC	CSR1_PR_AUC	TrainSec
21	0.9495	0.9559	0.9228	0.9334	140.0743
25	0.9631	0.9688	0.9360	0.9462	387.9208
31	0.9662	0.9698	0.9419	0.9469	479.9729

The sensitivity analysis shows that retaining more local mRNA context improves predictive performance on both the WT and independent datasets, with the best overall results obtained at the full 31-nt target-site length, albeit at increased computational cost.

### Experimental performance comparison with representative baselines

We have trained a set of baselines representing heuristic-based, feature-based, and deep learning-based models and rbpCNN on WT CLASH dataset using the same data split and evaluated the models on the independent CSR-1 CLASH dataset.

All baseline models implemented in this study were trained under a unified experimental protocol (same data splits, early stopping strategy, and comparable hyperparameter budget), while results from Yang et al.^32^ are reported as published and not re-implemented in the next section. We separate the baseline results that used the same experimental protocol (Table 9) and the results taken from Yang et al.^32^ (Table 10), and report them in separate tables.

**Table 9:** Comparison of rbpCNN with a set of representative baseline models.

Model	AUC	Accuracy	Precision	Recall	F1
rbpCNN	0.9419	0.8674	0.9374	0.7873	0.8558
DL_BiLSTM	0.7930	0.7238	0.7046	0.7705	0.7361
kmer_xgb	0.7806	0.7069	0.7136	0.6910	0.7021
DL_CNN	0.7689	0.6976	0.6926	0.7105	0.7015
kmer_rf	0.7408	0.6790	0.6789	0.6792	0.6790
DL_Transformer	0.7377	0.6675	0.6757	0.6439	0.6594
SeedMatch	0.6071	0.5007	0.5003	0.9999	0.6669
kmer_logreg	0.6062	0.5712	0.5724	0.5622	0.5673

**Table 10:** Performance comparison on an independent dataset **CSR-1 CLASH**^32^ which is not used for training and validation at all.

	F1	Precision	Recall	Accuracy	AUC
Baseline CNN by Yang et al.^1^	0.776	0.794	0.758	0.781^2^	0.866
Model by Yang et al.^1^ ^32^	0.857	0.887	0.830	0.862^2^	0.933
rbpCNN (Proposed Model)	0.8558	0.9374	0.7873	0.8674	0.9419

^1^Reported by Yang et al. ^32^. These values are taken directly from the original publication and are not re-implemented in this study.

^2^Calculated from reported F1, Precision and Recall values.

**Fig. 11:**
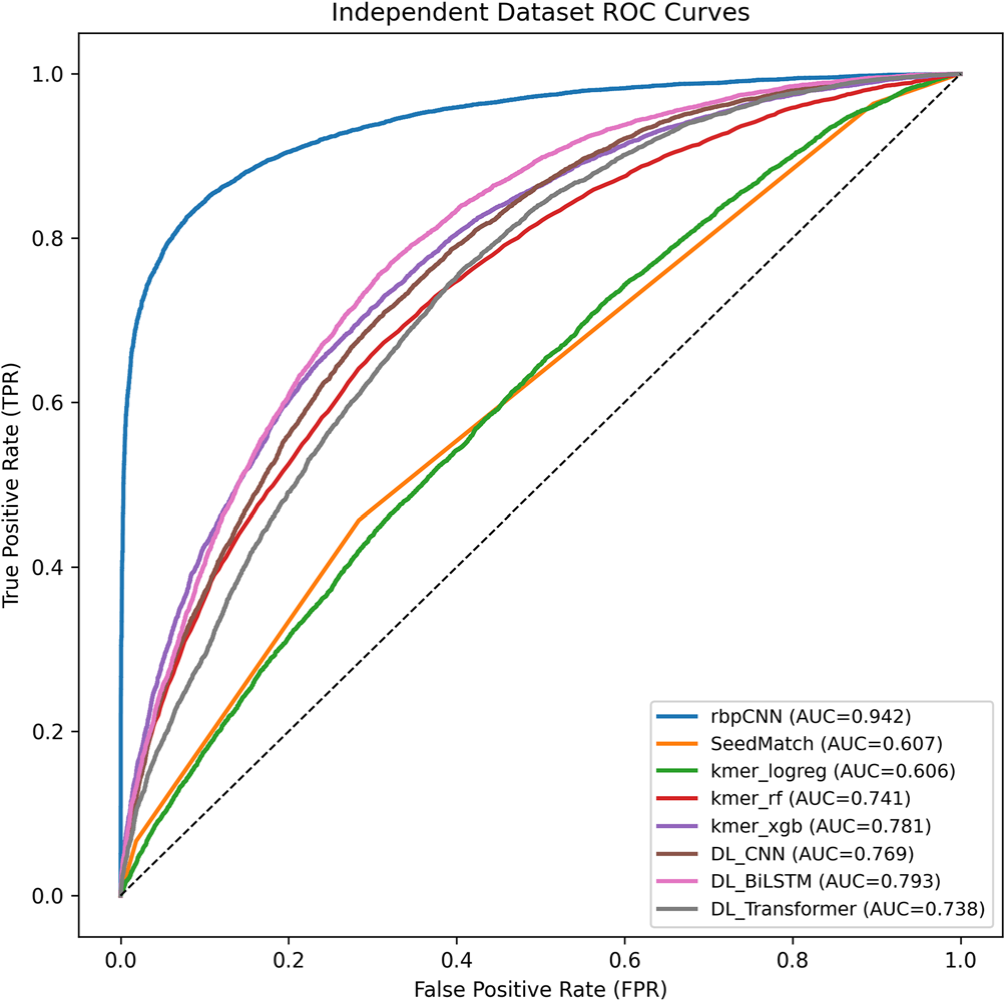
ROC-AUC Comparison of rbpCNN with baselines representing heuristic-based, feature-based, and deep learning-based models.

Figure 11 summarizes ROC–AUC performance on the independent CSR-1 CLASH test set. Across all operating points, rbpCNN exhibits a consistently higher true-positive rate at any fixed false-positive rate, yielding the best discrimination (AUC = 0.942). This large margin over alternative approaches indicates that modeling piRNA–mRNA interactions benefits from explicit interaction-map representations and multi-channel priors that encode pairing compatibility and local structural context, rather than relying solely on sequence composition or generic sequence encoders.

SeedMatch is a deterministic, knowledge-driven scoring heuristic that searches for short complementary “seed-like” matches between the piRNA and the candidate mRNA region. In practice, it slides a fixed-length window (e.g., 7 nt) along the sequences and returns a score proportional to the best match under canonical base-pairing constraints. As shown in Fig. 11, SeedMatch provides only limited ranking ability (AUC ≈ 0.607), producing a curve close to the diagonal. This behavior is expected: while seed-like pairing can capture a subset of strong interactions, it ignores broader context such as non-seed pairing, bulges/mismatches, positional effects along the site, and any secondary-structure accessibility effects; therefore, many true and false pairs obtain similar heuristic scores, reducing separability in ROC space.

We included a set of k-mer–based machine learning models that represent each piRNA–mRNA pair by bag-of-k-mer counts (or TF/IDF-like weighting), then learn a classifier on these fixed-dimensional features.

kmer_logreg (Logistic Regression) is a linear classifier over sparse k-mer features. It captures additive contributions of motifs but cannot model higher-order interactions between distant k-mers across the two sequences. Its performance (AUC ≈ 0.606) suggests that, on this independent set, purely linear motif evidence is insufficient for robust generalization and yields discrimination comparable to the simple heuristic baseline.

kmer_rf (Random Forest) aggregates many decision trees over k-mer features, enabling non-linear rules and feature interactions. This improves performance (AUC ≈ 0.741) relative to kmer_logreg, consistent with the idea that interaction prediction depends on non-linear combinations of sequence patterns.

kmer_xgb (Gradient-boosted trees) further enhances non-linear modeling capacity by sequentially correcting errors through boosting, typically yielding stronger ranking than bagged trees on high-dimensional sparse features. Correspondingly, it achieves the best ML baseline performance (AUC ≈ 0.781), approaching but still remaining clearly below neural baselines and far below rbpCNN.

Overall, the ML baselines demonstrate that learned motif composition and non-linear decision boundaries already provide meaningful predictive power; however, they remain limited by the representational bottleneck of k-mer counts, which discard spatial alignment, pairing geometry, and structured interaction patterns.

The DL baselines learn embeddings directly from the raw sequences using standard sequence encoders, and combine piRNA and mRNA representations for classification.

DL_CNN uses convolutional filters over nucleotide embeddings to detect local motifs and short interaction-related patterns. Convolutions can capture short-range dependencies efficiently but may struggle with long-range cross-sequence alignment unless explicitly designed for interactions. Its performance (AUC ≈ 0.769) is comparable to kmer_xgb, indicating that local motif detectors alone do not fully resolve the interaction discrimination problem.

DL_BiLSTM encodes sequences with bidirectional recurrent layers, capturing longer-range dependencies and sequential context. It provides the strongest DL baseline (AUC ≈ 0.793), suggesting that modeling context beyond local motifs improves generalization on CLASH-derived pairs.

DL_Transformer uses self-attention to model global dependencies and flexible token-to-token interactions within each sequence representation. The Transformer baseline was not exhaustively tuned, and its performance may be improved with more extensive architecture and training optimization. In this setup it attains AUC ≈ 0.738, slightly below BiLSTM and CNN. This outcome is plausible when training data size, regularization, and inductive biases favor simpler architectures; Transformers may require careful tuning (depth/heads, positional encoding, and training schedule) to outperform recurrent models in moderate-scale biological datasets.

While the DL baselines operate on sequences largely independently (then fuse representations), rbpCNN directly represents the pair as an interaction tensor, enabling the network to learn discriminative patterns that depend on explicit cross-sequence alignment and pairing structure. This interaction-centric inductive bias is reflected in the ROC curve: rbpCNN maintains high TPR even at low FPR, implying fewer false positives when operating in stringent regimes—an important property for downstream experimental validation where controlling false discoveries is critical. Quantitatively, rbpCNN improves AUC by ~ 0.15 over the best DL baseline and by ~ 0.16–0.34 over the ML baselines, demonstrating that the proposed architecture’s explicit modeling of interaction structure provides a decisive advantage in independent-set generalization.

The separation between curves is most pronounced in the low-to-moderate FPR range (approximately 0–0.2), where rbpCNN achieves a steep rise in TPR. This regime is typically the most relevant for biological screening: a method that can retrieve a large fraction of true interactions while keeping the false-positive rate low reduces both experimental cost and interpretational noise. In contrast, heuristic SeedMatch and linear k-mer scoring behave close to random ranking, underscoring that piRNA–mRNA interaction prediction—particularly under CLASH conditions—is not well captured by seed heuristics or linear motif evidence alone.

Table 9 reports a comparison between rbpCNN and representative baselines trained under the same protocol. The proposed rbpCNN achieves the strongest performance across all reported metrics in this benchmark, achieving the highest AUC (0.9419) and F1-score (0.8558), which highlights its strong discriminative capability and balanced precision–recall trade-off. Among deep-learning baselines, the BiLSTM model performs best (AUC = 0.7930), indicating that modeling sequential context improves over convolution-only and Transformer encoders, although these generic architectures still lag well behind rbpCNN. The k-mer–based machine-learning models show moderate performance, with gradient boosting (kmer_xgb) outperforming random forests and logistic regression, reflecting the benefit of non-linear feature interactions but also the limitations of fixed k-mer representations that ignore explicit interaction structure. In contrast, the rule-based SeedMatch baseline yields a low AUC (0.6071), indicating weak ranking ability; its extremely high recall arises because the heuristic assigns positive scores to nearly all pairs that contain any short complementary region, leading to most samples being predicted as positive under a fixed threshold. Overall, the table demonstrates that explicitly modeling piRNA–mRNA interaction patterns, as done in rbpCNN, is critical for achieving robust and well-balanced prediction performance.

**Fig. 12:**
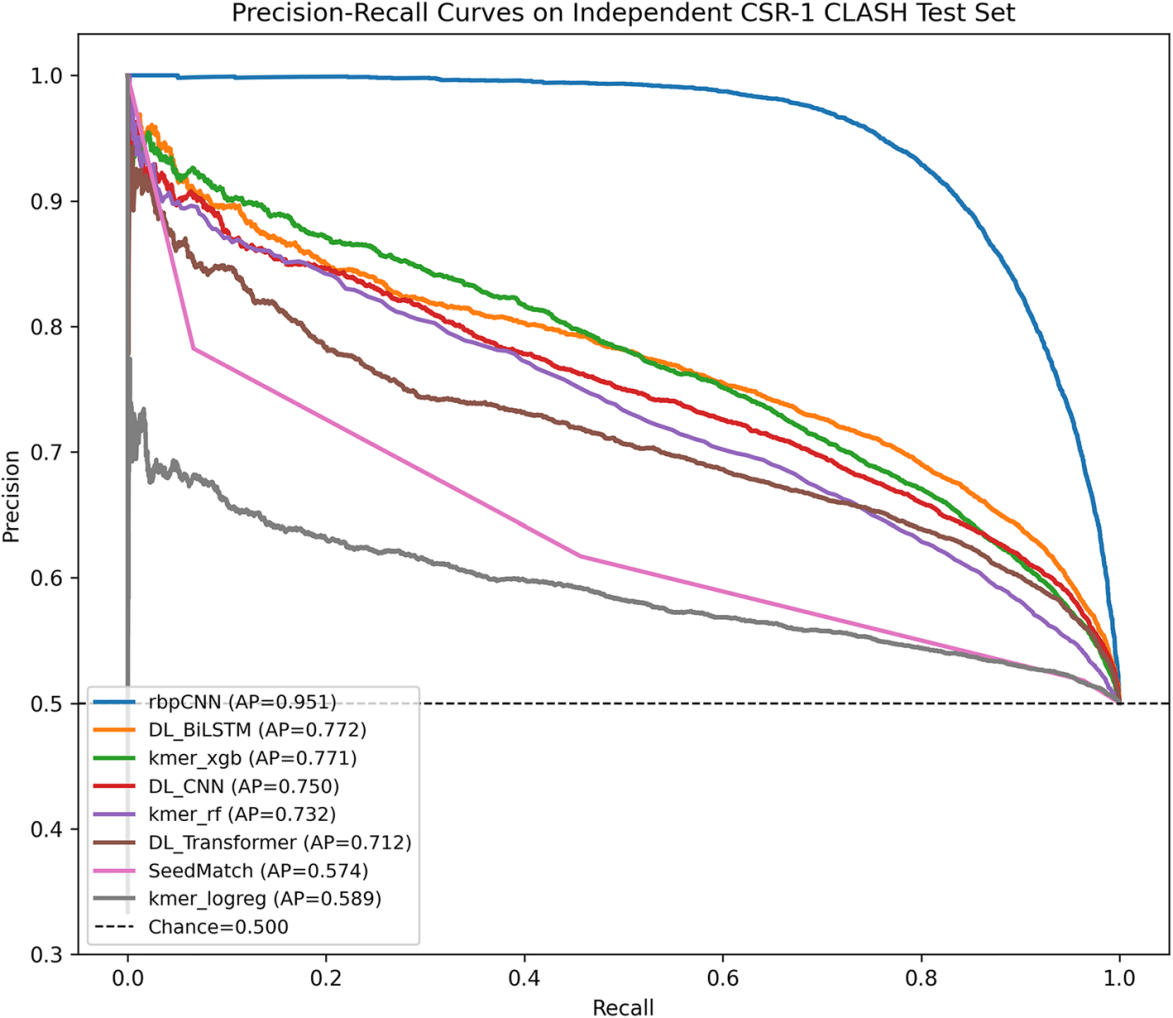
Precision-Recall Curve and Average Precision (AP) Comparison of rbpCNN with baselines representing heuristic-based, feature-based, and deep learning-based models.

Figure 12 depicts that the Precision–Recall analysis on the independent CSR-1 CLASH test set further confirms the strong discriminative ability of rbpCNN. As shown in the PR curve, rbpCNN achieves the highest Average Precision (AP = 0.951), with a clear margin over all competing methods, and maintains very high precision across almost the entire recall range. Among the baselines, DL_BiLSTM (AP = 0.772) and kmer_xgb (AP = 0.771) provide the strongest alternative performance, followed by DL_CNN (AP = 0.750), kmer_rf (AP = 0.732), and DL_Transformer (AP = 0.712). In contrast, the simpler heuristic and linear baselines, SeedMatch (AP = 0.574) and kmer_logreg (AP = 0.589), perform substantially worse and remain much closer to the chance level. Overall, these results indicate that rbpCNN not only ranks positives more accurately in ROC space, but also preserves a much better precision–recall balance on the independent dataset, which is particularly important in biological screening scenarios where false positives are costly.

### Performance comparison with reported results of models in literature using the same dataset

**Fig. 13:**
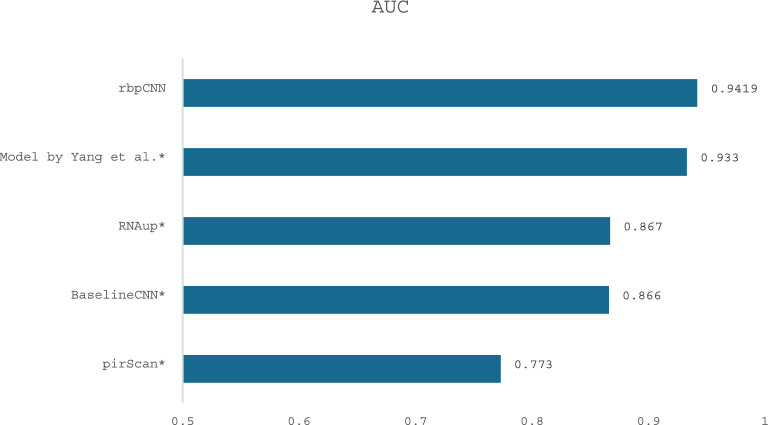
AUC Comparison of rbpCNN with Deep Learning (DL) (Model by Yang et al. and BaselineCNN) and non-DL models (RNAup^22^ and pirScan^37^) (Data marked by * are reported by Yang et al.^32^).

The results shown in Table 10 and Fig. 13 obtained on the CSR-1 CLASH independent dataset indicate that the proposed rbpCNN model achieves stronger overall discrimination than the baseline CNN and the attention-based model reported by Yang et al.^32^, and two other models tested on the same dataset, RNAup^22^ and pirScan^37^.

The rbpTransformer^33^ comparison is not included in Table 10 because that study used CSR-1 CLASH^32^ as an additional dataset for training and validation rather than as a fully independent external test set. rbpTransformer obtains an AUC of 0.9438 and an accuracy of 0.859 on the WT CLASH dataset using k-fold validation. rbpCNN performs better in k-fold validation with an AUC of 0.9655 and an accuracy of 0.9066.

In this section, we use reported values from related papers in the literature. Accordingly, literature-based comparisons should be interpreted as benchmark-level reference points rather than as fully controlled head-to-head re-implementations.

In this study, we use the small CSR-1 CLASH dataset as an entirely unseen dataset to further validate the results. This same approach is followed by Yang et al.^32^ for their model and the baselines they use for comparison. So, in this section, we compare our model to these approaches in Table 10 and Fig. 13.

On the CSR-1 CLASH dataset (Table 10), rbpCNN achieves the highest AUC (0.9419), exceeding the baseline CNN (0.866) and Yang et al.’s model (0.933). Notably, while Yang et al.’s model reports slightly higher recall (0.830 vs. 0.7873), rbpCNN compensates for this with a significant increase in precision (0.937 vs. 0.887), leading to the best balance between precision and recall (F1 = 0.856) and the highest overall accuracy (0.867). These results indicate that rbpCNN is particularly effective at reducing false positives, which is crucial for reliable piRNA–mRNA interaction prediction. We note that we did not perform a threshold search to improve accuracy, F1, recall, and precision on the independent dataset. The threshold is taken as 0.5 for consistency and to avoid post-hoc threshold optimization on the independent dataset.

We note that Yang et al. achieve better recall on CSR-1, while rbpCNN demonstrates greater precision and AUC. This points to a distinctive operating point on the precision-recall trade-off: rbpCNN takes a more cautious approach in identifying positives, which minimizes false positives (crucial when downstream validation is expensive) but results in the omission of some true interactions. Notably, AUC indicates the quality of ranking across different thresholds, and rbpCNN’s superior AUC implies enhanced separability even if the standard threshold leads to lower recall.

Figure 13 compares the ROC–AUC results between rbpCNN and both deep-learning and traditional (non-DL) baselines. rbpCNN achieves the highest AUC of 0.9419, surpassing Yang et al.’s deep model (0.933) and the BaselineCNN by Yang et al. (0.866), which does not include the proposed biophysics-guided channel augmentation. Notably, rbpCNN also outperforms non-DL methods RNAup (0.867) and pirScan (0.773), representing thermodynamics/accessibility-based and rule-based scoring, respectively. The AUC increase over BaselineCNN (+ 0.0759) shows that the improvement is mainly due to the additional channels that incorporate interaction-informed priors into the input, not just the CNN backbone. Additionally, the margin over RNAup (+ 0.0749) indicates that rbpCNN effectively combines coarse structural/accessibility signals with learned spatial patterns to better identify true interactions.

Overall, the comparisons indicate that rbpCNN provides strong performance relative to existing approaches, with particularly favorable AUC and precision on the evaluated benchmark, while also generalizing effectively to unseen datasets.

## Discussion

In this study, we introduced a biophysics-informed Interaction-CNN framework to predict piRNA–mRNA binding interactions with improved robustness and generalization. Compared to earlier methods, several key improvements were made.

First, the input representation was expanded to a 21-channel tensor that combines interaction identity, structural compatibility, helix-run detectors, positional deltas, and Nussinov-derived unpaired profiles. This richer representation enables the model to utilize both sequence composition and secondary-structure constraints, creating a biologically grounded feature space.

Second, to reduce overfitting often seen in previous deep learning models, we added stronger dropout regularization, weight decay (L2 penalty), and an adaptive early-stopping strategy. These changes significantly narrowed the performance gap between training and validation data, as shown in the stable learning curves.

Third, to promote reproducibility and efficiency, we implemented persistent caching of Nussinov unpaired vectors, preventing repeated computations and maintaining consistent structural features across folds and evaluations.

The results show that the proposed model achieves high predictive accuracy, with strong performance in accuracy, F1-score, and area under the ROC curve (AUC) across cross-validation folds and on an independent CSR-1 CLASH dataset. Notably, the model strikes a balance between sensitivity and specificity, indicating it effectively captures biological signals rather than dataset artifacts.

The independent testing further demonstrates the model’s ability to generalize beyond training data, which is crucial for applications in piRNA functional annotation and target identification.

Despite these improvements, some limitations remain. While the Nussinov algorithm offers a simple and computationally efficient approximation of RNA secondary structure, it does not fully reflect the complexity of RNA thermodynamics and tertiary interactions. Using more accurate structure prediction methods, like minimum free energy folding or ensemble-based approaches, could further boost predictive power.

Although our model combats overfitting with stronger regularization, it may still be affected by class imbalance and dataset biases. Strategies such as data augmentation, contrastive learning, or transfer learning across different organisms could enhance robustness.

We have used k-fold splitting for the WT CLASH dataset. While a grouped/cluster-based split could further reduce possible sequence overlap across folds, the external CSR-1 CLASH evaluation—kept entirely unseen during model development—already serves as an out-of-distribution test supporting the model’s generalization.

Additionally, the current framework mainly depends on sequence and structure data; integrating multi-omics information, such as expression levels, epigenetic marks, or Argonaute protein binding profiles, might offer a more comprehensive view of piRNA–mRNA interaction landscapes.

Future research could explore explainable AI techniques (e.g., saliency maps, attention visualization) to identify sequence motifs or structural patterns most responsible for predictions. This would improve interpretability for experimental validation and help uncover new biological principles behind piRNA targeting. Furthermore, extending the model to multi-task learning, where piRNA–mRNA interactions are predicted along with related tasks like binding affinity estimation or subcellular localization, could leverage shared representations and enhance generalization.

A limitation is that the structural channel currently uses a classic maximum-base-pairing model (Nussinov) rather than thermodynamics-based folding. Future work can systematically evaluate ViennaRNA/RNAstructure-derived accessibility signals (e.g., unpaired probabilities from the partition function) using the same cross-validation protocol to quantify the effect of the choice of folding backend.

Another limitation of the current input design is that the model operates on a fixed local target window. Thus, very distal or long-range pairing patterns outside the selected 31-nt region are not explicitly represented.

## Conclusions

In this work, we developed a biophysics-informed Interaction-CNN framework for predicting piRNA–mRNA binding interactions. By integrating sequence-derived interaction maps with structural priors through a unified 21-channel representation and Nussinov-based unpaired profiles, the proposed rbpCNN effectively captures key biological aspects of piRNA–mRNA recognition. The incorporation of robust regularization strategies, including dropout, weight decay, and adaptive early stopping, proved essential for controlling overfitting and ensuring strong generalization. Comprehensive evaluation through cross-validation on the WT CLASH dataset and independent testing on the CSR-1 CLASH dataset demonstrates that rbpCNN consistently achieves high discriminative performance across multiple metrics. In particular, on the independent CSR-1 CLASH dataset, which was not used during training or validation, rbpCNN achieves performance that is competitive with, and in several metrics exceeds, previously reported results on the same dataset. These findings indicate that explicitly modeling interaction-level features offers a clear advantage over sequence-only and heuristic-based approaches under identical data conditions. Although a precision–recall trade-off is observed, this behavior is expected in realistic binding-prediction tasks and can be flexibly adjusted via threshold calibration to meet different application requirements. Overall, the results establish rbpCNN as a robust and reliable framework for large-scale piRNA target prediction, with performance comparable to or superior to existing approaches evaluated on the same benchmark. Beyond predictive accuracy, the interaction-centric design of rbpCNN provides a biologically grounded foundation that facilitates improved generalization and interpretability. Future work may focus on incorporating advanced RNA structure prediction techniques, integrating multi-omics data, and applying explainable artificial intelligence methods to further enhance biological insights and discovery.

## Data Availability

Reference rbpCNN Python Code and used datasets are available at [https://github.com/AhmetGurhanli/rbpCNN](https:/github.com/AhmetGurhanli/rbpCNN).
